# RNA-binding protein CELF2 inhibits breast cancer cell invasion and angiogenesis by downregulating NFATc1

**DOI:** 10.3892/etm.2022.11170

**Published:** 2022-01-27

**Authors:** Limin Zhou, Xiju Xie

Exp Ther Med 22:Article no. 898, 2021; DOI: 10.3892/etm.2021.10330

Subsequently to the publication of the above article, an interested reader drew to the authors’ attention that an error was made during the compilation of Fig. 2. Specifically, in Fig. 2A, the same image for the mice (accurately presented for the ‘OverExp-CELF2’ experiment) was inadverently chosen to also represent the ‘Con’ experiment.

The authors have re-examined their raw data and identified the data that should have been included in the figure. The corrected version of Fig. 2 is shown on the next page, now including the correct data for the ‘Con’ experiment in Fig. 2A. Note that this error did not have a major impact on either the overall results or on the conclusions reported in this study. The authors regret the error that was made during the assembly of Fig. 2, and apologize to the readership for any inconvenience caused.

## Figures and Tables

**Figure 2:**
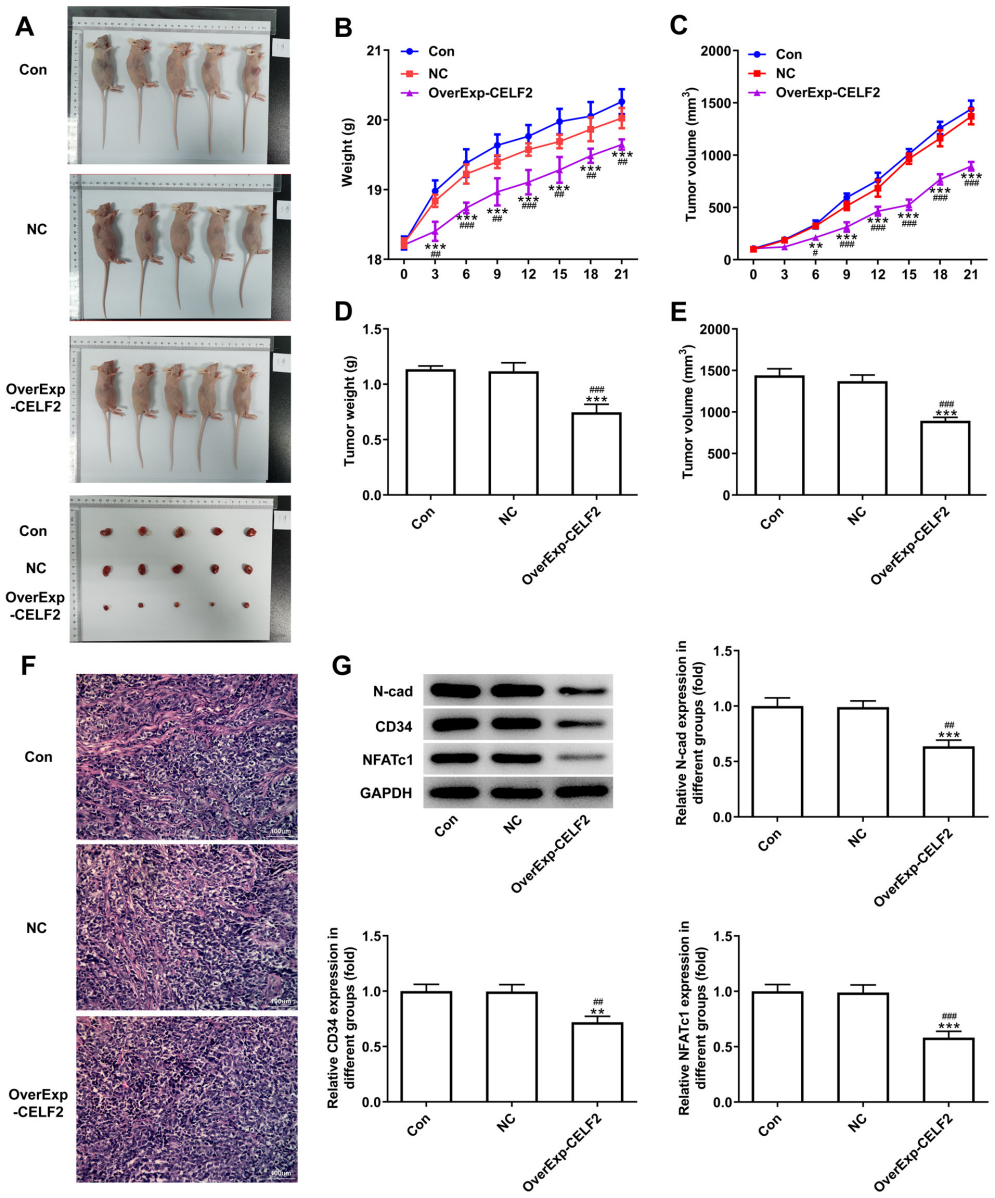
CELF2 overexpression inhibits tumor growth and angiogenesis. (A) Mice and tumor in the three groups. (B) Mouse weight changed from day 0 to day 21. ^***^P<0.001 vs. Con group. ^##^P<0.01 and ^###^P<0.001 vs. NC group. (C) Tumor volume changed from day 0 to day 21. ^**^P<0.01 and ^***^P<0.001 vs. Con group. ^#^P<0.05 and ^###^P<0.001 vs. NC group. (D) Tumor weight at day 21. ^***^P<0.001 vs. Con group. ^###^P<0.001 vs. NC group. (E) Tumor volume at day 21. ^***^P<0.001 vs. Con group. ^###^P<0.001 vs. NC group. (F) Angiogenesis was analyzed by HUVEC tube formation assay; scale bar, 100 µm. (G) Western blot analysis was performed to detect the expression of proteins associated with invasion and angiogenesis and NFATc1 in tumor tissues. ^**^P<0.01 and ^***^P<0.001 vs. Con group. ^##^P<0.01 and ^###^P<0.001 vs. NC group. CELF2, CUGBP Elav-like family member 2; NFATc1, nuclear factor of activated T cells 1; Con, control; NC, negative control.

